# Optimization of a One-Step Heat-Inducible *In Vivo* Mini DNA Vector Production System

**DOI:** 10.1371/journal.pone.0089345

**Published:** 2014-02-20

**Authors:** Nafiseh Nafissi, Chi Hong Sum, Shawn Wettig, Roderick A. Slavcev

**Affiliations:** School of Pharmacy, University of Waterloo, Waterloo, Ontario, Canada; Federal University of São Paulo, Brazil

## Abstract

While safer than their viral counterparts, conventional circular covalently closed (CCC) plasmid DNA vectors offer a limited safety profile. They often result in the transfer of unwanted prokaryotic sequences, antibiotic resistance genes, and bacterial origins of replication that may lead to unwanted immunostimulatory responses. Furthermore, such vectors may impart the potential for chromosomal integration, thus potentiating oncogenesis. Linear covalently closed (LCC), bacterial sequence free DNA vectors have shown promising clinical improvements *in vitro* and *in vivo*. However, the generation of such minivectors has been limited by *in vitro* enzymatic reactions hindering their downstream application in clinical trials. We previously characterized an *in vivo* temperature-inducible expression system, governed by the phage λ *pL* promoter and regulated by the thermolabile λ CI[Ts]857 repressor to produce recombinant protelomerase enzymes in *E. coli*. In this expression system, induction of recombinant protelomerase was achieved by increasing culture temperature above the 37°C threshold temperature. Overexpression of protelomerase led to enzymatic reactions, acting on genetically engineered multi-target sites called “Super Sequences” that serve to convert conventional CCC plasmid DNA into LCC DNA minivectors. Temperature up-shift, however, can result in intracellular stress responses and may alter plasmid replication rates; both of which may be detrimental to LCC minivector production. We sought to optimize our one-step *in vivo* DNA minivector production system under various induction schedules in combination with genetic modifications influencing plasmid replication, processing rates, and cellular heat stress responses. We assessed different culture growth techniques, growth media compositions, heat induction scheduling and temperature, induction duration, post-induction temperature, and *E. coli* genetic background to improve the productivity and scalability of our system, achieving an overall LCC DNA minivector production efficiency of ∼90%.We optimized a robust technology conferring rapid, scalable, one-step *in vivo* production of LCC DNA minivectors with potential application to gene transfer-mediated therapeutics.

## Introduction

**Figure 1:**
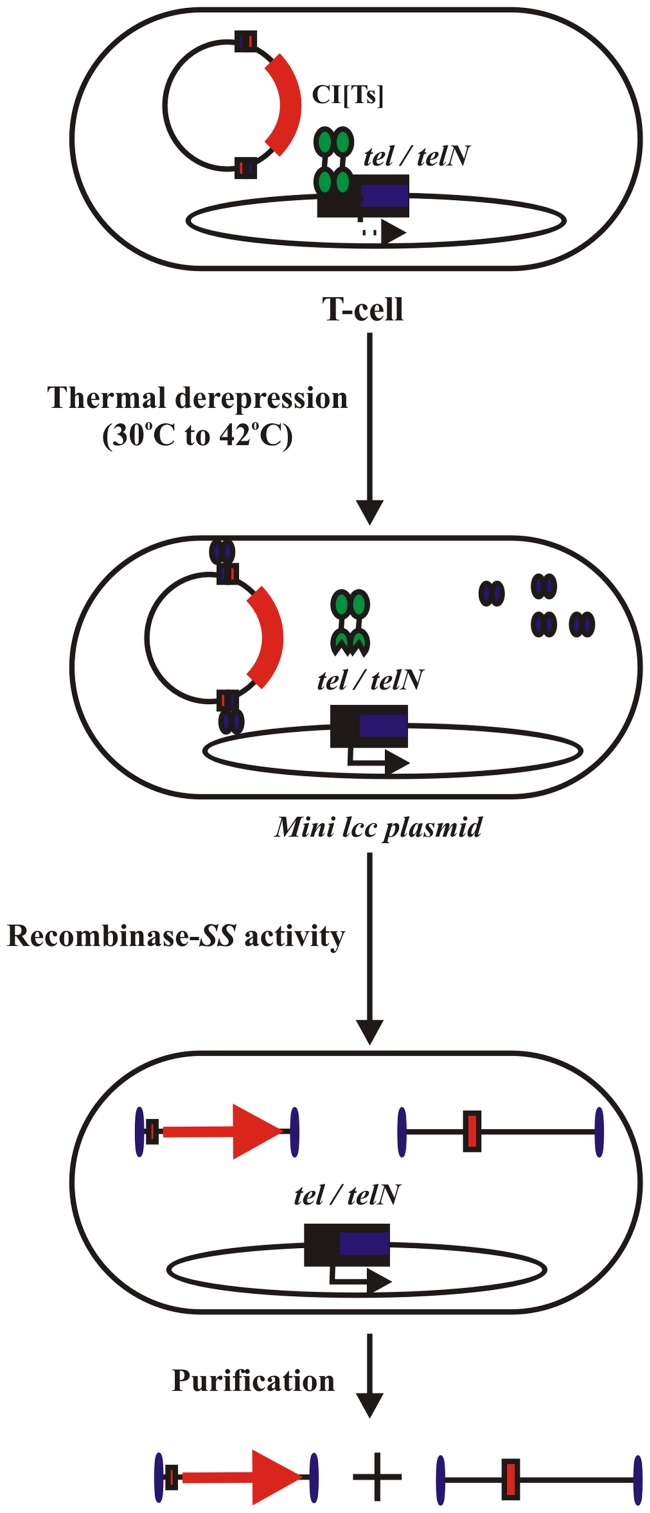
Conditional processing of the parent plasmid DNA vectors by site-specific recombination. Tel^+^ (W3NN) cell conditional processing of parent pNN9 plasmid into DNA ministrings. Under induced conditions, W3NN cells lead to the production of LCC DNA ministrings by Tel protelomerase activity on its target site encoded within the two Super Sequence sites on pNN9.

Temperature inducible expression systems, consisting of bacteriophage λ strong *pL* and/or *pR* promoters under the regulation of the thermo-labile CI[Ts]857 repressor were exploited to thermally regulate recombinase expression in a linear covalently closed (LCC) DNA minivector production system. This temperature-sensitive expression system has been widely used for the production of various recombinant proteins in prokaryotic cells. Successful DNA minivector production *via* this one-step system was previously demonstrated, achieving expression of recombinant bacteriophage PY54, N15, and P1-derived recombinase enzymes, Tel, TelN, and Cre, respectively upon increasing culture temperatures above 37°C [1]. However, temperature up-shifts to 40°C or higher, along with the recombinase over-expression, impart various intracellular stress responses and enhanced plasmid DNA (pDNA) replication rates. In this study, we sought to optimize our novel one-step *in vivo* DNA minivector production system by genetic and process engineering. We assessed DNA ministring production efficiency under various temperature schedules and growth conditions in combination with genetic modifications influencing pDNA vector replication, processing rates, and cellular heat stress responses. We noted that culture growth techniques, temperature shift scheduling, and specific genetic knock-outs, each served to improve parental plasmid processing efficiency into LCC DNA minivectors. We have previously described an *in vivo* DNA minivector production system, whereby vectors are the result of an *in vivo* site-specific recombination process [1]. The parental pDNA carries the gene of interest (GOI) expression cassette, flanked by two multi-target sites called “Super Sequences” (SS), and the bacterial sequences encoded in the plasmid backbone that are necessary for the maintenance and propagation of plasmids in bacterial cells. The *in vivo* expression of the respective recombinases resulted in the excision of the recognition site, dividing the parental circular covalently closed (CCC) plasmid into two smaller molecules: 1) a LCC mini-plasmid carrying the bacterial backbone sequences, and 2) a LCC DNA minivector carrying the GOI expression cassette (Fig. 1) [1].

The recombinases exploited to generate bacterial-sequence-free DNA vectors, in the forms of supercoiled circular covalently closed (CCC) DNA, are derived either from the tyrosine family of recombinases including, but not limited to, λ integrase (Int) and P1 derived Cre and Flp from the yeast plasmid 2-µm circle; or the serine family such as the highly efficient *Streptomyces* bacteriophage ΦC31-derived integrase [2], [3]. The phage N15 and PY54-derived recombination systems, involving protelomerases, also separate the GOI expression cassette from the parental CCC pDNA backbone in the form of linear DNA with closed ends, referred to as linear covalently closed (LCC) DNA ministrings [1]. These vectors serve to improve both transfection efficiency and safety [1, unpublished data].

In this study, the strong λ *pR* promoter, governed by the temperature-sensitive CI857 repressor, was employed to construct a temperature-inducible expression system whereby high level expression of the Tel protelomerase occurs under temperature-induced conditions but not under repressed conditions. Our approach to integrate a single copy of the protelomerase gene under the control of the λ expression system into the *E. coli* chromosome produced an effective but imperfect plasmid processing efficiency similar to related studies that obtained a maximum of 50–90% CCC minivector recovery according to agarose gel electrophoresis (AGE) and ethidium bromide (EtBr) staining.

The *E. coli* K-12 host has been selected for this study due to its widespread application, relative ease of modification and management, well-studied use in the production of a wide breadth of recombinant proteins, and potential cost-effective scalability. Additional factors favoring the selection of *E. coli* as the expression host of choice are the availability of a variety of strong inducible promoters and its well-recognized suitability for the maintenance and propagation of various pDNA vectors [4], [5]. Constitutive expression of a recombinant protein in the bacterial host results in high stress levels on cells because the metabolic load of recombinant protein production remains constant as the source of nutritional factors and energy become limited; this negatively impacts the growth rate of the host cell. In many situations, the best conditions for cell growth are different from those for recombinant protein production. As such, stringent control on the expression of protelomerase is required to impart efficient plasmid processing. First, the expression system must efficiently repress protelomerase expression prior to induction in order to avoid premature plasmid processing that would reduce plasmid propagation. Most promoters commonly employed for recombinant protein expression require the addition of an inducer molecule, the depletion or addition of a nutrient, or a shift in a physical or physicochemical factor, such as pH [6]. In theory, each of these options is practical and can promote efficient control in small laboratory settings. However, in large-scale settings suitable for commercial production of the final product, each option may present processing disadvantages. Chemical inducers, such as isopropyl β-D-1-thiogalactopyranoside (IPTG) or antibiotics, introduce additive processing costs and can be toxic at industrial level quantities, imposing additional costs for containment and elimination [7], [8]. Additional controls and downstream operations may also be required to remove chemical inducers, particularly from pharmaceutical-grade products intended for clinical application. This not only complicates bioprocessing, but can also dramatically increase cost of manufacture [9]. High level recombinant protein production may impart nutrient fatigue, whereby exhaustion of an amino acid results in starvation that compromises cell metabolism, recombinant protein synthesis, and/or plasmid replication [10]. This drawback could also be exacerbated by the challenges associated with the precise control of the induction duration in large-scale cultures. At present, very few pH-inducible expression systems are available for exploitation, characterization studies are still insufficient, and pH-mediated induction of gene expression can interfere with the optimal pH for recombinant protein folding, function, and physiological conditions [11]. In consideration of these system limitations, a thermo-regulated expression system has been employed to express the phage-derived protelomerase to process the parent plasmid into LCC minivectors. Thermo-regulated expression systems circumvent many of the drawbacks aforementioned including the use of special media, toxicity or expensive chemical inducers; these systems also possess several inherent advantages including ease of handling, minimized contamination risks, and scalability. Our system of choice employs the bacteriophage λ strong *pL* promoter, regulated by the thermolabile λ CI[Ts]857 repressor that has been extensively exploited for the regulated production of many recombinant proteins and peptides [12]. This system is based on inserting the GOI into different vectors that contain the strong major leftward (*pL*) promoter. The gene cloned downstream of the λ promoter is efficiently regulated by CI857, with an increasing expression profile as temperature rises from 30°to 40°C [13] and expression is increasingly repressed at temperatures below 37°C (normally in the range of 28–32°C for full suppression). Optimal repressor activity quickly diminishes at 37°C and becomes completely abrogated at temperatures above 37°C, permitting efficient transcription of the GOI by host RNA polymerase [14], [15]. However, temperatures above 40°C, that confer full depression and high level gene expression, also induce an adverse host metabolic environment for recombinant protein production. Heat induction triggers the heat shock response (HSR) that is controlled by the alternative heat shock sigma factor, σ^ 32^ (*rpoH* gene product) [16], [17]. The σ^ 32^ factor alters the expression of different genes, including transcription factors, by delivering RNA polymerase to gene promoters that comprise the heat shock regulon; it regulates the activity of the transcriptional apparatus itself and executes various roles in cell homeostasis [18]. The σ^32^ governed heat shock regulon is formed by a large family of genes, particularly those encoding heat shock proteins (HSPs) involved in protein folding and degradation. These include chaperones such as ClpB, DnaK/J and GroEL/S and proteases such as Lon, ClpP, ClpC, HsIV (ClpY) HsIU, ClpQ, and FtsH [19]. The HSR includes a rapid and selective synthesis of heat shock proteins (HSPs) soon after heat induction. This is followed by an adaptation period characterized by a lower rate of protein synthesis that eventually reaches a new steady-state level. In addition to the synthesis of HSPs following heat shock, the physiological response of *E. coli* includes the temporary decrease in growth rate, which directly impacts replication and segregation of pDNA. Among many different variables that may affect the heat-inducible systems, we sought to improve plasmid copy number and Tel protelomerase-mediated LCC DNA minivector production efficiency *via* our *in vivo* λ CI857/*pL* system, by optimizing culture growth techniques, growth media composition, induction scheduling, host cell genotype, and the use of a fluoroquinolone antibiotic, ciprofloxacin, that inhibits bacterial DNA synthesis.

## Results

### Plasmid copy number influences efficiency of parent plasmid processing to LCC DNA products

The DNA minivector production system involved thermo-regulated protelomerase expression accompanied by subsequent protelomerase-mediated processing of parental CCC pDNA into LCC DNA minivectors. The first step in optimizing this process was to examine the effect of parental pDNA copy number on the productivity of the CI857/*pL* system in generating the LCC DNA products. To do so, the high copy pNN13 vector (Table 1; 500–700 copies per cell) and medium copy pNN8 vector (30–100 copies per cell) were assessed for plasmid processing following protelomerase induction. Following extraction, DNA purity and integrity were checked by spectrophotometry and restriction analysis. DNA constructs with purity of A_260/280_ = 1.9–2 and _A260/230_>2 were selected for further investigations. AGE and restriction analysis was employed to assess DNA ministring production efficiency from parental pDNA. In all cases, the application of medium copy number plasmids, carrying the SS, exhibited a remarkably improved LCC DNA production efficiency. Interestingly, medium copy plasmids not only exhibited improved production efficiency, but also attained greater total LCC DNA yields compared to high copy plasmids that offered a higher amount of parental plasmid substrate (data not shown).

**Table 1:** Bacterial strains used in this study.

Strain	Genotype	Source
JM109	F', Δ *(gpt-lac)0*, *glnV44*(AS), λ*^−^*, *rfbC1*, *gyrA96*(NalR), *recA1*, *endA1*, *spoT1*?, *thi-1*, *hsdR17*, pWM5, F128-x	New England Biolabs
W3110	F-, λ*^−^*, *IN(rrnD-rrnE)1*, *rph-1*	CGSC #4474; [50]
W3110-Cre (W1NN)	F-, λ*^−^*, *IN(rrnD-rrnE)1*, *rph-1 lacZ::Cm-cI857-cre*	[1]
W3110-TelN (W2NN)	F-, λ*^−^*, *IN(rrnD-rrnE)1*, *rph-1 lacZ::Cm-cI857-telN*	[1]
W3110-Tel (W3NN)	F-, λ*^−^*, *IN(rrnD-rrnE)1*, *rph-1 lacZ::Cm-cI857-tel*	[1]
JW0427	F-, *Δ(araD-araB)567, ΔlacZ4787(::rrnB-3), ΔclpP723::kan, λ-, rph-1, Δ(rhaD-rhaB)568, hsdR514*	*E. coli* Genetic Stock Center (CGSC)#8590
JW0429	F-, *Δ(araD-araB)567, ΔlacZ4787(::rrnB-3), Δlon-725::kan, λ-, rph-1, Δ(rhaD-rhaB)568, hsdR514*	CGSC#8592
JW4131	F-, *Δ(araD-araB)567, ΔlacZ4787(::rrnB-3), λ-, rph-1, Δ(rhaD-rhaB)568, ΔhflX723::kan, hsdR514*	CGSC#10974
JW4132	F-, *Δ(araD-araB)567, ΔlacZ4787(::rrnB-3), λ-, rph-1, Δ(rhaD-rhaB)568, ΔhflK724::kan, hsdR514*	CGSC#10975
BW25113	F-, *Δ(araD-araB)567, ΔlacZ4787(::rrnB-3), λ-, rph-1, Δ(rhaD-rhaB)568, hsdR514*	CGSC #7636
BW25113-Tel	F-, *Δ(araD-araB)567, ΔlacZ4787(::rrnB-3), λ-, rph-1, Δ(rhaD-rhaB)568, hsdR514, lacZ::Cm-cI857-tel*	This Study
JW0427-Tel (Δ*clpP*)	F-, *Δ(araD-araB)567, ΔlacZ4787(::rrnB-3), ΔclpP723::kan, λ-, rph-1, Δ(rhaD-rhaB)568, hsdR514,* Δ*clpP*	This Study
JW0429-Tel (Δ*lon*)	F-, *Δ(araD-araB)567, ΔlacZ4787(::rrnB-3), ΔclpP723::kan, λ-, rph-1, Δ(rhaD-rhaB)568, hsdR514,* Δ*lon*	This Study
JW4131-Tel (Δ*hflX*)	F-, *Δ(araD-araB)567, ΔlacZ4787(::rrnB-3), ΔclpP723::kan, λ-, rph-1, Δ(rhaD-rhaB)568, hsdR514,* Δ*hflX*	This Study
JW4132-Tel (Δ*hflK*)	F-, *Δ(araD-araB)567, ΔlacZ4787(::rrnB-3), ΔclpP723::kan, λ-, rph-1, Δ(rhaD-rhaB)568, hsdR514,* Δ*hflK*	This Study

### Heat induction schedules strongly influence the production efficiency of LCC DNA products

**Figure 2:**
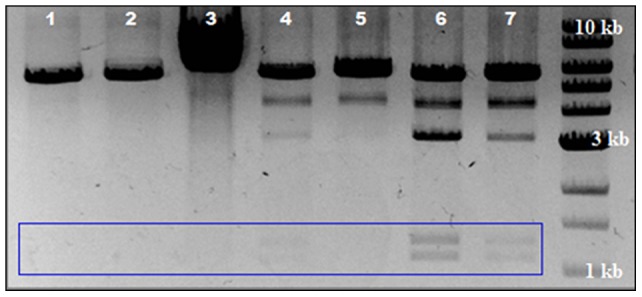
Effect of duration of heat induction in CI857/*pL*/*pR* system on Tel protelomerase activity. Following heat induction at 42°C for a series of induction durations, Tel^+^ (W3NN) cells, harboring the parent plasmid (pNN9), were returned to either a fully (30°C) or partially repressed (37°C) state. LCC DNA ministrings were extracted from cells and digested by a single cutter restriction enzyme and an equal quantity of extracted DNA was analyzed by AGE and EtBr staining. The larger single band (5.6 kb) represents residual CCC parental plasmid; while the medium band (3.2 kb) represent the LCC backbone DNA and the two smaller bands (purple box) represent successfully processed DNA ministring vector. **Lane 1**) uninduced control; **L2**) 15 min induction back to 30°C; **L3**) 15 min induction back to 37°C; **L4**) 30 min induction back to 30°C; **L5**) 30 min induction back to 37°C; **L6**) 60 min induction back to 30°C; **L7**) 60 min induction back to 37°C; and **L8**) 1 kb DNA ladder.

**Figure 3:**
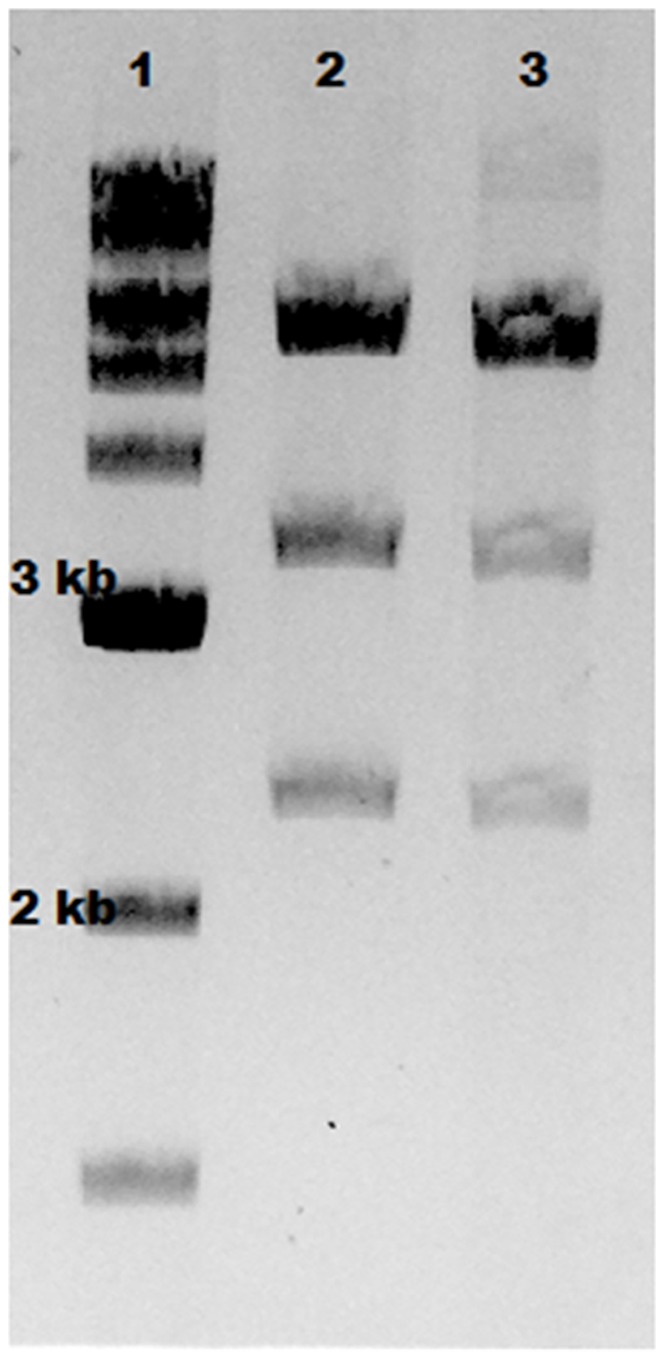
Effect of fast or slow temperature shift in CI857/pL/pR system on Tel protelomerase activity. Tel^+^ (W3NN) cells, harboring the parent plasmid (pNN9), were grown to A_600_ = 0.8 and exposed to fast or slow temperature upshift from 30°C to 42°C followed by subsequent incubation at 42°C for 30 min before fast or slow temperature downshift to fully repressed conditions at 30°C. DNA vectors were extracted from cells and an equal number of extracted DNA was analyzed by AGE and EtBr staining. The larger single band (5.6 kb) represents residual circular parent plasmid, while the medium bands represent the processed LCC backbone DNA and LCC DNA ministring vector. From the left: 1 kb DNA ladder; **Lane 1**) gradually shifted; **L2**) rapidly shifted.

Various heating strategies were employed to examine the effects of induction duration and LCC DNA minivector production by minimizing the adverse effects of temperature up-shift on growth rate, cell damage, decreased viability and productivity, and plasmid instability. We assayed a series of heat induction durations from 5 to 60 min with Tel^+^ wild type (W3NN) cells carrying the parental pDNA, W3NN [pNN9], at mid-log phase and post-induction temperature at either fully repressed (30°C) or partially repressed (37°C) for 4 h. We found that LCC DNA production was highest when using a schedule where cells were induced for 60 min before returning to fully repressed conditions (Fig. 2). Interestingly, partial repression was not as effective when returning 60 min induced cells to 37°C and shorter induction periods of 15 or 30 min did not provide adequate protelomerase induction regardless of the subsequent resting temperature. As expected, the non-induced control conferred no observable LCC DNA conversion. We also exposed cells to fast or slow temperature up-shift for protelomerase expression in order to assess the productivity of our system during a temperature change and generally noted that cells subjected to a gradual temperature up-shift adapted to thermal stresses better than those exposed to a fast temperature increase (Fig. 3).

### A two stage continuous growth approach improves LCC DNA production efficiency and scalability

**Figure 4:**
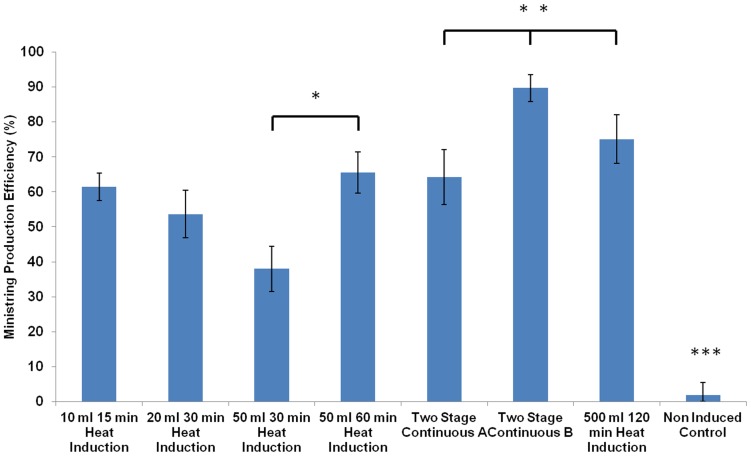
Effect of culturing technique and volume on ministring production. LCC DNA ministring production efficiencies of Tel^+^ (W3NN) cells for: 1) heat induced batch cultivations across different culture volumes (10, 20, 50 ml) and heat induction duration (15, 30, 60 min); 2) heat induced two stage continuous cultivations (A & B); 3) 500 ml batch cultivation; and 4) non-induced controls. For 50 ml cultures, heat induction for 60 min led to production efficiencies significantly greater than heat induction for 30 min * (P<<0.01). Production efficiencies for two stage continuous (B) was significantly greater than two step continuous (A) ** (P<<0.01) and 500 ml batch cultivation ** (P<0.05). All heat induced cultures had production efficiencies significantly greater than non-induced samples *** (P<<0.01).

We next explored various induction schedules with the intention of maximizing protelomerase expression, corresponding to high LCC DNA product yields upon maximal protelomerase-mediated conversion from the parental pDNA. The duration of temperature up-shift was optimized in order to avoid the detrimental effects associated with prolonged heat shock. Increasing culture volumes required a corresponding increase in the duration of temperature up-shift such that expression could be effectively induced. Cultivation under repressed (30°C) conditions resulted in high density cultures while maintaining plasmid stability. Subsequent transition into the induced state at 42°C led to the high protelomerase expression necessary for processing to LCC DNA. Experimental conditions mimicking the two-stage continuous culturing strategy were examined in small-scale laboratory settings for evaluation against batch cultivation. Thermo-regulated control of protelomerase expression was confirmed as non-induced cultures yielded significantly lower LCC DNA production compared to all induced conditions (P<<0.01; Fig. 4). The LCC DNA production from 10, 20, and 50 ml batch cultivations required corresponding increases in duration of heat induction (15, 30, and 60 min) for optimal production efficiencies. Such a trend was made obvious by significant differences in LCC DNA products retained between 30 min (38.0%) and 60 min (65.5%) heat induction in 50 ml cultures (P<<0.01). The lower production efficiencies were denoted by greater amounts of parental pDNA, signifying insufficient protelomerase expression and protelomerase-mediated LCC DNA production. Between batch and two-stage continuous cultivation, LCC DNA production with two-stage continuous culture (A) resulted in efficiency of 64.2% while batch cultivation, using equivalent 500 ml culture volumes and similar induction durations of ∼60 minutes, did not yield any LCC DNA products (data not shown). Two-stage continuous culture with an additional hour of heat induction (B) resulted in a significant increase in LCC DNA production efficiency (89.7%) when compared to two-stage continuous (A) (P<<0.01) and 500 ml batch cultivation with 120 min of heat induction (P<0.05). In absolute terms per extraction, LCC DNA minivector yields were approximated to be 27.5 µg, 87 µg, and 150 µg for two stage continuous (A), 500 ml batch cultivation, and two stage continuous (B) respectively. This corresponds to an estimated 3.2 pg, 12.1 pg and 20.5 pg of LCC DNA minivector produced per bacterial cell for each of the three above listed variations.

### LCC DNA production is enhanced in a *ΔhflX* host mutation background

**Figure 5:**
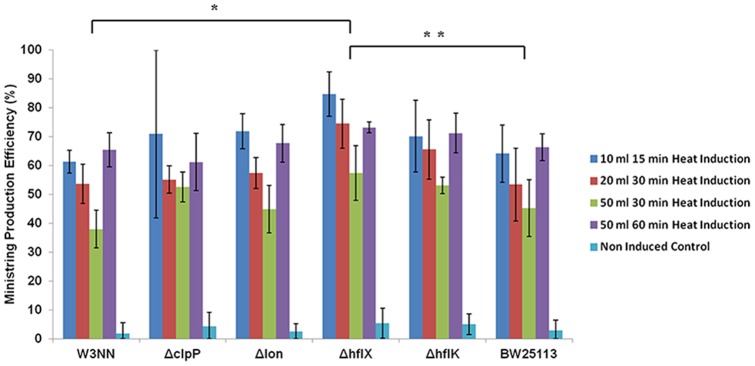
Effect of protease mutation on DNA ministring production efficiency. LCC DNA ministring production efficiencies were assessed at various culture volumes and heat induction durations for strains possessing various protease associated gene deletions (Δ*clpP,* Δ*lon,* Δ*hflx,* Δ*hflk*) and isogenic Tel^+^ strain (BW25113). Across all conditions, the introduction of *hflX* gene deletion conferred significant increases in ministring production efficiencies over the original Tel^+^ wild type cells (W3NN) carrying the parent plasmid (pNN9) * (P≤0.01) and isogenic Tel^+^ cells (BW25113) carrying pNN9 ** (P<0.05).

Induced overproduction of recombinant proteins contribute to metabolic aberrations and stresses and may result in numerous cellular responses including general stress, heat shock, stringent, and SOS responses; such responses may elicit altered physiological states including a growth arrest and a decrease in specific growth rates. As such, we hypothesized that protelomerase turnover time may be compromised at elevated temperatures due to protease-mediated degradation, thereby reducing the processing efficiency of parental pDNA into LCC DNA minivectors. To test the effects of proteases and associated gene products on LCC DNA production efficiency, we assessed parental plasmid processing outcomes in a variety of protease and protease-associated isogenic mutants (Fig. 5). Similar trends associated with thermo-regulated expression of protelomerase across all strains were observed as efficiency of conversion to LCC DNA products was significantly lower (P<0.05) in non-induced samples, compared to induced samples at all culture volumes. In correlation with results from Tel^+^ wild type W3NN [pNN9], LCC DNA production from 10, 20, and 50 ml batch cultivations required identical increases during heat induction (15, 30, and 60 min) for optimal LCC production efficiency. Across all conditions, the introduction of an *hflX* gene deletion conferred a significant increase in LCC DNA production efficiency compared to the wild type W3NN (P≤0.01) and isogenic wild type BW25113 (P<0.05) counterparts, with ideal production seen in 10 ml cultures after 15 min *tel* heat induction.

### Enriched media and ciprofloxacin influences LCC DNA production efficiency

**Figure 6:**
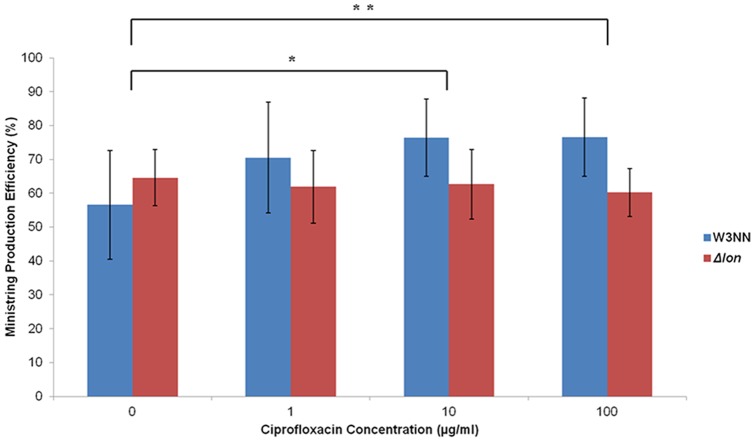
Ministring DNA production efficiency upon the introduction of ciprofloxacin at various concentrations. Application of 0, 1, 10 and 100 µg/ml of ciprofloxacin added to wild type Tel^+^ cells (W3NN) and Δ*lon* derivative harboring the parental pNN9 plasmid. Addition of 10 and 100 µg/ml of ciprofloxacin added to Tel^+^ cells conferred a 19.8% * (P<0.10) and 20.0% ** (P<0.10) increase in DNA ministring production efficiency compared to the untreated induced control. Increases to ministring production efficiency were not observed for Δ*lon* derivative.

We attempted to synchronize plasmid replication rates with protelomerase expression to enhance the efficiency of LCC DNA production in our one-step *in vivo* system. To do so, we hypothesized that transient inhibition by the fluoroquinolone antibiotic, ciprofloxacin, would elicit diminished plasmid replication which may correspond to improved LCC DNA production efficiency. The Δ*lon* derivative was of particular interest as it was speculated to curb the metabolic burden associated with SOS response triggered by ciprofloxacin, reducing the already heavy metabolic load dedicated to the overexpression of recombinant protelomerase. The application of ciprofloxacin to Tel^+^ W3NN cells harboring the CCC pDNA precursor plasmid [pNN9] led to a gradual, but not statistically significant, increase in LCC DNA processing efficiency from about half to 76.6% (Fig. 6). In contrast, the application of ciprofloxacin to the Δ*lon* derivative did not induce any change in production efficiency. We also investigated whether culture media could impact precursor pDNA processing to LCC DNA and found that conversion efficiency was highest in cells growing in an enriched culture media, where protelomerase expression was induced when cells were in late log growth phase. Minimal M9 media proved to be the poorest at enabling LCC DNA production, even with an additional carbon source added prior to induction (data not shown).

## Discussion

We previously reported the application of bacteriophages N15 and PY54 recombination systems to the development of an *in vivo* system for production of LCC DNA minivectors [1]. We inserted a single copy of the PY54 *tel* gene into the *E. coli* genome to avoid some of the drawbacks of overproduction and episomal expression of the protelomerase as well as resultant stresses, metabolic imbalances, and/or accumulation of recombinant protelomerase in inclusion bodies post induction [20]. During prolonged periods of temperature up-shift, the propagation of plasmid-free cells is favored since plasmid partitioning and stable plasmid inheritance at high temperature is not particularly efficient, since approximately 50% of the dividing cells lose the plasmid after prolonged temperature shifts to 42°C. However, this complication can be alleviated by incorporating selective antibiotics into growth media [12] or by inserting the recombinant protein gene and its CI857*/pL/pR* regulatory cassette into the bacterial genome, thereby circumventing issues of plasmid inheritance. For large-scale production systems, where the absence of antibiotics or other selective chemicals is preferred, the latter approach would be favored. Furthermore, maintenance and replication of the residing plasmid overloads the host metabolic pathways and consumes important cellular resources. In our case, such metabolic burden and energetic drain is exacerbated by the induction of Tel protelomerase enzyme at late logarithmic growth phase [6].

We found that the medium-copy pDNA vector, offering 20–100 copies per cell, conferred greater LCC DNA production efficiency compared to its high-copy (hundreds per cell) counterpart. We could dramatically increase the productivity of our system to yield higher quantities of LCC DNA conversion by using a continuous growth production system with tight control over heat induction cycles and constant fresh media supplementation. Improved recombinant protein expression systems employing a CI857-regulated heat induction system using continuous growth or fed-batch systems compared to batch systems has previously been shown [21]. Fed-batch systems and two continuous cultures connected in series have also been shown to maximize cell concentration and further improve expression of the recombinant enzyme, while avoiding plasmid loss [22].

Induction temperature, induction duration, and the specific growth rate pre- and post-induction are critical factors that can be controlled and optimized to improve the productivity of thermal inducible systems [23]. We demonstrated the highest LCC DNA production using a schedule whereby cells were grown at a repressed state to late log phase before inducing for 60 min at 42°C and then returned to a fully repressed state at 30°C. This contrasts an earlier study, where the assessment of different induction schedules for the production of recombinant human growth hormone, in a fed batch fermentation system, denoted greatest recombinant protein expression after 20 min at 42°C and observed protein degradation after 40 min [24]; varying thermostabilities of recombinant protelomerase and human growth hormone was speculated to contribute to such differences.

The LCC DNA production system offers several advantages that enable optimal generation of LCC DNA products. Chromosomal integration of the protelomerase gene cassette permits higher constraints on protelomerase expression in addition to thermoregulated controls achieved *via* the CI[Ts]857 repressor. Integration of the protelomerase gene cassette ensures gradual protelomerase expression upon heat induction that would not likely be conferred by a multicopy episomal plasmid expression system.

Chromosomal expression of the *tel* protelomerase served to mediate the negative effects associated with dual stresses of heat shock and rapid recombinant protein production. Since the expressed Tel protelomerase enzyme was capable of acting on multiple copies of the target plasmid, high LCC DNA production efficiencies can be attained with relatively moderate enzyme expression. This attribute contributed to reducing the duration of heat induction required for protelomerase expression and optimal LCC DNA production. Previous studies have shown diminished cell growth upon 1–2 hours of heat induction, at 42°C, due to metabolic burden associated with recombinant protein production, the expression of heat shock proteins and reduced translation activities; this also resulted in slight decrease in biomass concentration and reduced accumulation of recombinant proteins beyond two hours following heat induction [25]. It was interesting to note that the high production efficiencies achieved by two stage continuous cultivation method was accomplished approximately two hours following initial heat induction. From the experimental findings, optimal LCC DNA production was achieved by heat induction at durations with high recombinant protelomerase expression, prior to cessation of cell growth and other negative effects associated with prolonged heat shock.

In all instances of induction, a gradual temperature increase from 30°to 42°C was applied as previous findings indicated an observed improvement in recombinant protein expression with slowest heating rates when compared to more rapid rates. We found that rapid heating rates resulted in decreased recombinant protein expression, likely due to the high ATP demand associated with rapid recombinant protein production and expression of heat shock proteins. Transcriptional analysis by Caspeta *et al*. indicated a dramatic increase in mRNA levels for heat shock genes and such increases were most significant at rapid heating rates that contributed to high ATP demands as a result of the activation of cellular responses to mediate the rapid onset of heat stress. The high ATP demand caused an imbalance between glycolytic and TCA cycles, shifting ATP production *via* cellular respiration to glycolysis, and led to increases in substrate level phosphorylation and the generation of acetate by-product [25]. Correlations between rapid heating rates and rapid onset of heat stress is likely supported by the corresponding increase in *rpoH* mRNA levels imparted by more rapid heating rates [25]. In contrast, slower heating rates allow cells to better accommodate for heat induction and dampen the negative effects associated with rapid heat shock. These findings may be applicable in large-scale production of LCC DNA vectors since slow heating rates are generally attained in large-scale fermenters.

Induced overproduction of recombinant enzymes contributed to metabolic aberrations and stresses triggering numerous cellular responses including general stress response, heat shock-like response, stringent response and SOS response, where such responses may elicit altered physiological states including: 1) growth arrest and a decrease in specific growth rates [26]; 2) alterations to central carbon metabolism [27]; 3) ribosome degradation and diminishing protein synthesis [28]; and 4) protein misfolding/degradation [29], [30]. As overexpressed recombinant enzymes often require folding modulators, such high level expression may overload translational machinery resulting in protein misfolding. As such, the potential for misfolding increases with the utilization of strong promoters and high levels of induction [31]. The accumulation of unfolded/misfolded proteins favors protein aggregation, inclusion body formation and the activation of the RpoH regulon, triggering a heat shock-like response. The RpoH regulon with at least 120 genes conferred the expression of HSPs including chaperones (ClpB, DnaK/J, GrpE GroEL/ES, ibpA, ibpB) and proteases (FtsH, Lon, ClpP, DegP, OmpT) that are associated with protein folding and degradation [29], [32]–[34]. High level expression down-regulated translational machinery and diverted resources away from cell growth/division and the formation of various aggregate sizes and complexity poses interference to normal cellular metabolism [35]. These combined effects become significantly more prominent under nutrient-depleted conditions, found in high density cultures. High cell density and recombinant enzyme production constitutes nutrient-limiting and slow growth conditions that introduce general stress response and subsequent entry into stationary phase upon activation of the RpoS regulon [26], [29], [36], [37].

Simultaneously, heat induction activates the HSR, affecting the quantity and overall quality of the recombinant enzyme. Temperature induction negatively affects protein folding and increases inclusion body formation [26], [38]. The activation of heat shock proteases leads to the degradation of recombinant protein. Recombinant proteins were more susceptible to proteolytic degradation mediated by five ATPase associated with diverse cellular activities (AAA+) heat shock proteases Lon, ClpYQ/Hs1UV, ClpAP, ClpXP, and FtsH [30], [31], [37], [39]. Previous studies had shown an upregulation of folding chaperones (DnaJ, DnaK, GroEL, ClpB), small heat shock proteins (sHSP) (IbpA, IbpB), and proteases (Lon, FtsH) upon dual stresses of heat shock and high level recombinant protein production [25], [40]. In this study, protease-specific and associated gene deletions were introduced in an attempt to promote optimal protein re-folding by folding chaperones such that protein degradation by heat shock proteases would be minimized and theoretically improve expression and extend protein turnover of functional protelomerases, and subsequently enhance LCC DNA processing efficiency. Contrary to our initial hypothesis, gene deletions of *clpP, lon* and *hflK* did not result in any significant improvements. Lack of observed differences in production efficiency can be attributed to the fine control of protelomerase expression and the minimal heat induction necessary for optimal protelomerase-mediated LCC DNA production. Short durations of heat induction applied in this study did not result in high amounts of recombinant protein that severely overburdened the activity of folding chaperones – a common observation seen in traditional recombinant protein production systems. As such, the activities and impact of heat shock proteases became less prominent in affecting LCC DNA production since folding chaperones effectively re-folded majority of misfolded proteins and prevented the formation of non-functional proteins or inclusion bodies. Nevertheless, slight improvements in production efficiencies were observed upon the deletion of *lon* and *hflK* gene across all culturing and heating conditions. Improvements in LCC DNA production for Δ*lon* derivatives were speculated to be attributed to diminished degradation of small heat shock proteins, IbpA and IbpB, by Lon protease [41]. IbpA and IbpB are holding chaperones that intercalated into protein aggregates and stabilize folding intermediates for folding chaperones [31], [34], [38]. The Δ*lon* derivative should impart reduced IbpA and IbpB degradation and improved protein re-folding. HflKC, a heterodimer consisting of HflK and HflC, have been shown to modulate the proteolytic activity of FtsH protease. It was previously demonstrated that the deletion of *hflKC* genes resulted in reduced degradation of cytosolic CII protein and enhanced the degradation of membrane bound SecY protein [42]–[45]. Hence, the deletion of *hflK* gene was expected to confer decreased degradation of cytosolic recombinant Tel protelomerase leading to the slight improvement in LCC DNA processing efficiency.

We also noted a greater level of variability of LCC DNA production efficiency, particularly for culture volumes of 10 ml and 50 ml with 15 min and 60 min heat induction, respectively. This may be explained by previous work that reported a suppression of general stress response, mediated by RpoS, during strong overexpression of recombinant proteins [46]. Deletion of the *clpP* gene, expressing the ClpP protease that specifically cleaves RpoS, was shown to negatively impact recombinant protein production due to greater sigma factor competition as result of higher RpoS stability [46]. For thermoregulated systems, higher stability of RpoS may outcompete RpoH for RNA polymerase and negatively affect the accumulation of heat shock proteins necessary to effectively respond against heat stress [25]. Influences of higher RpoS stability in Δ*clpP* cells under dual stress of heat shock and high levels of recombinant protelomerase production may contribute to highly variable efficiencies in LCC DNA processing. A statistically significant improvement in LCC DNA processing was observed in the Δ*hflX* derivative, potentially due to the interactions between HflX and ribosomal subunits. Activities of HflX likely serve to regulate and control protein translation rates in co-ordination with the down-regulation of translation related genes and reduced concentrations of ribosomal components upon heat stress and recombinant protein production [25], [46], [47]. As such, the deletion of the *hflX* gene may prompt higher rates of protein translation and protelomerase expression, improving LCC DNA production efficiency.

## Conclusion

The results of this study served as an optimal protocol to significantly improve LCC DNA vector processing efficiency in our novel *in vivo* mini DNA vector production system. We constructed, characterized, and optimized a robust technology by applying bacteriophage PY54-derived recombination system to construct an *in vivo E. coli* platform for the time/cost effective production of LCC DNA minivectors. We anticipate that this novel technology will offer valuable application toward the generation of new DNA vectors in health, agriculture and other industries.

## Methods

### Strains and plasmids

*E. coli* K-12 strains were used to generate all recombinant cell constructs and JM109 were particularly employed as hosts for plasmid constructions and amplification. A list of bacterial strains and plasmids used in this study are shown in Table 1 and Table 2.

**Table 2:** Plasmid vectors used in this study.

Plasmid	Genotype	Source
pBRINT	*lacZ::cat-MCS::lacZ* (Cm^R^)	NBRP; [51]
pGL2	*SV40P-Luc-PolyA-SV40 intron*	Promega
pUC57-SS	*Multi-target site “SS”* (Ap^R^)	GenScript
pcDNA5/FRT	*Flp-In Integrating vector frt-hyg-pCMV::MCS-BGHpA* (Ap^R^ Hyg^R^)	Invitrogen
pNN7	*pGL2-egfp switched for luc*	[1]
pNN8	*pNN7+ SS (upstream of SV40 promoter)*	[1]
pNN9	*pNN8-SS (2XSS) (second SS downstream of SV40 polyA sequence)*	[1]
pNN12	*pBRINT* (Cm^R^) *(SS^+^)*	This study
pNN13	*pcDNA5/FRT (SS^+^)*	This study

### Construction of recombinant Tel Cells

W3110 was used to generate W3NN Tel^+^ cells representing the *in vivo* platform for production of bacterial sequence depleted LCC DNA vectors. These cells were employed for heat-inducible *in vivo* Tel protelomerase expression as previously described [1].

### Construction and linearization of high and medium copy pDNA vectors in recombinant cells

The multi-purpose target site, named Super Sequence (SS), was designed in our group as previously explained, synthesized by GenScript and cloned into the pUC57 by *Eco*RI and *Hin*dIII. To construct modified DNA vectors, the SS fragment was moved from pUC57 into the MCS of pBRINT (Cm^R^) vector (National Bioresource Project; NBRP) by *Bam*HI and *Eco*RI to generate pNN12 and moved from pNN12 by *Bam*HI and *Xho*I into MCS of the high copy pcDNA5/FRT vector (Invitrogen) to produce pNN13 (5.35 kb). The medium copy plasmid vector pNN8 (5.3 kb) was constructed as previously described [1]. New constructs were tested and confirmed by colony PCR and analytical digestion. W3NN cells were transformed with 0.1 μg of pNN8 and pNN13 DNA constructs and selected on LB agar + ampicillin (Ap) (50 μg/ml). A single colony was grown overnight in 5 ml LB agar + Ap (50 μg/ml) in repressed conditions at 30°C with aeration. A 1:100 dilution of fresh cells were prepared in 10 ml and grown in a 250 ml flask at 30°C with aeration to mid-log phase A_600_ = 0.6. Protelomerase expression was induced upon temperature up-shift and incubation at 42°C for 30 min during mid-log phase of bacterial growth, before downshifting to 30°C overnight. Cells were then harvested and plasmid extracted (Omega kit, VWR). LCC DNA production was assayed by AGE and digestion by *Nde*I. Standard recombinant DNA cloning and transformation techniques were performed as described by [48].

### Assessing the quality of the DNA minivectors

Bacterial sequence free LCC DNA minivectors, extracted from induced bacterial cells, were checked for purity using a NanoDrop spectrophotometer. The ratio of the absorbance at 260 and 280 nm (A_260/280_) was used to assess the purity of DNA minivectors from proteins and the absorbance at 260 and 230 nm (A_260/2–230_) to assess the purity from RNA. DNA constructs showing A_260/280_ of 1.9–2.0 and A_260/230_>2.0 were selected for restriction analysis and AGE. Equal quantities of extracted DNA were analyzed by AGE to check the integrity of the extracted DNA minivectors.

### Assessing effects of volume and culturing conditions on the production efficiency of LCC DNA products

For batch cultivation, a single colony of W3NN [pNN9] was grown overnight in 5 ml LB + Ap (100 µg/ml) under repressed conditions at 30°C with aeration. Fresh cells were grown from the overnight culture at 1∶100 dilution of various culture volumes (10, 20, 50, 500 ml) in a 250 ml or 2 L Erlenmeyer flask at 30°C with aeration to late log phase A_600_ = 0.8. Protelomerase expression was induced upon temperature up-shift and incubation at 42°C for various timed durations (15, 30, 60, 120 min). After induction, the cultures were subjected to various post-induction temperatures either at 30°C or 37°C for 4 h prior to overnight incubation at 30°C. Cells were harvested and plasmid extracted with *E.Z.N.A.* Plasmid Mini/Maxi-Prep Kit (Omega, VWR). LCC DNA processing efficiency was assessed by AGE and densitometry (AlphaImager, Alpha Innotech). All LCC DNA production efficiencies were calculated as a percentage of the total amount of the two LCC DNA product species over the total amount of monomeric pDNA comprising of LCC DNA and all isoforms of the parental plasmid.

For representative two-stage continuous cultivation, a single colony was grown overnight in 5 ml LB + Ap (100 µg/ml) under repressed conditions at 30°C with aeration. Two batches of fresh cells were grown from the overnight culture at 1∶100 dilution of 50 ml LB + Ap in 250 ml Erlenmeyer flasks at 30°C with aeration to late log phase A_600_ = 0.8. Cells were then collected, centrifuged at 4K RPM for 10 min, and re-suspended in 1 ml of LB + Ap. The re-suspensions were added into a preheated 2L Erlenmeyer flask containing 500 ml of LB + Ap (100 µg/ml) for incubation at 42°C until A_600_ = 1.0 (A); an alternative heating condition was assessed where temperature up-shift was extended for an additional hour after A_600_ = 1.0 (B). Cultures were subjected to gradual temperature downshift and grown at 30°C overnight. Cells were harvested and plasmid extracted with *E.Z.N.A.* Plasmid Maxi-Prep Kit (Omega, VWR). DNA ministring production was assessed by AGE and densitometry (AlphaImager, Alpha Innotech). Calculations for the approximate amount of DNA ministring in each extraction was accomplished through densitometry comparisons between the 2.4 kb band and the 3 kb band of the DNA ladder, a known standard containing 125 ng of DNA for every 10 µl of DNA ladder, along with subsequent extrapolations accounting for the amount of sample loaded and the total eluted volume from each respective extraction. The estimated bacterial cell concentration in the extracted culture was calculated according to optical density readings from A_600_.

### Assessing the introduction of protease associated gene deletions for enhancing LCC DNA processing efficiency

The protelomerase expression cassette (*lacZ::cat-cI857-tel* (Cm^R^)) from the previously developed mini DNA vector production system (W3NN) was introduced into four different strains, conferring four protease associated single gene knockouts (Δ*clpP,* Δ*lon,* Δ*hflx*, Δ*hflk*) [49], and their parental strain by P1 bacteriophage-mediated transduction. W3NN cells were grown overnight in 5 ml LB + Cm (25 µg/ml) at 30°C. Phage generation from the donor strain was accomplished by the addition of 1∶10 dilutions of P1rev6, prepared in 1 ml of TN buffer (0.01 M Tris-HCl and 0.1 M NaCl, pH 7.8, Fisher Scientific, USA), into 300 µl of W3NN before adding 3 ml of top agar (Bacto Tryptone and Bacto Agar from Difco Laboratories, Sparks, MD). The solution was poured onto LB plates and incubated for 24 h at 37°C prior to lysate preparation. Approximately 5 ml of TN buffer was added to the surface of the plate and incubated for an additional 8 h at 4°C. TN buffer and the underlying top agar were collected into a sterile conical tube and centrifuged at 12 K RPM (Avanti J-E Centrifuge, Beckman Coulter, Mississauga, Canada) at 4°C for 20 min. Lysate was poured into a new pre-chilled conical tube and briefly vortexed upon the addition of 50 µl of CHCl_3_ (Fisher Scientific, USA). The five recipient strains were grown overnight in 5 ml LB or 5 ml LB + Kan (50 µg/ml) at 30°C prior to transduction. 1 ml of each of the overnight cultures was centrifuged at 6000 RPM for 5 min; the supernatant was discarded and the pellet was re-suspended with 1ml of 10 mM MgSO_4_+10 mM CaCl_2_ salt solution. 100 µl of the cell re-suspension was mixed with 100 µl LB and 100–1000 µl of phage lysate depending on phage titre. The cell mixture was incubated at 37°C for 20 min to allow phage adsorption and infection. Subsequently, 200 µl of 1 M sodium citrate and 1 ml of LB were added prior to 1.5–2 h incubation at 30°C. Cells were centrifuged at 6 K RPM for 5 min and re-suspended in 10 µl of 1M sodium citrate and 90 µl of LB; the complete cell re-suspension was plated on LB + Cm (25 µg/ml) using the spread plate technique. Plates were incubated at 30°C for 24–48 h and colonies were re-plated on new LB + Cm (25 µg/ml) plates supplemented with sodium citrate. Standard transformation techniques were performed as described by [48]. Heat induction using batch cultivation was performed as previously mentioned.

### Assessing effects of bacterial culture media and bacterial growth rate on LCC DNA production

Fresh overnight W3NN [pNN9] cells were diluted 100-fold in 10 ml of minimal M9 media (1X M9 salt composed of mixed solution of the (NH_4_)_6_Mo_7_O_24,_ H_3_BO_3_, CaCl_2_, 1 mM MgSO_4_, 1 µM CaCl_2,_ 0.2% Carbon source, pH 7.0), or enriched LB media (1.0% Tryptone, 0.5% Yeast Extract, 1.0% NaCl, pH 7.0). Cells were grown in a 50 ml flask at 30°C with aeration to reach corresponding phases of bacterial growth A_600_ = 0.2, 0.4, or 0.8. To induce protelomerase expression and plasmid processing, W3NN [pNN9] cells were subjected to temperature upshift and incubation at 42°C for 30 min before downshifting to 30°C overnight. Cells were then harvested and extracted by the *E.Z.N.A.* Mini-Prep Kit (Omega, VWR). LCC DNA production was assessed by AGE and restriction analysis.

### Assessing effects of ciprofloxacin on plasmid replication and cell division on LCC DNA production

A single colony of W3NN [pNN9] and Δ*lon* [pNN9] was grown overnight in 5 ml LB + Ap (100 µg/ml) under repressed conditions at 30°C with aeration. Fresh cells were grown from the overnight culture at 1∶100 dilution of 50 ml LB + Ap (100 µg/ml) in a 250 ml Erlenmeyer flask at 30°C with aeration until late log phase of bacterial growth (A_600_ = 0.8). Protelomerase expression was induced upon temperature up-shift and incubation 42°C for 60 min. After induction, the 50 ml cultures were divided into four 10 ml batches followed by the addition of ciprofloxacin at different concentrations (0, 1, 10, 100 µg/ml). The different batches were then incubated at 37°and 30°C for 30 and 60 min respectively prior to plasmid extraction by *E.Z.N.A.* Plasmid Mini-Prep Kit (Omega, VWR). LCC DNA production was assessed by AGE and densitometry (AlphaImager, Alpha Innotech).

### Statistical analysis

Welch's T test was used as statistical analysis to compare production efficiencies of tested samples with controls. Production efficiencies for all tested samples and controls were repeated and averaged with a minimum of three trials (N≥3).
